# Medication for Opioid Use Disorder Service Provision and Telephone Counseling: A Concurrent Mixed-Methods Approach

**DOI:** 10.3390/ijerph18116163

**Published:** 2021-06-07

**Authors:** Rosemarie Martin, Augustine W. Kang, Audrey A. DeBritz, Mary R. Walton, Ariel Hoadley, Courtney DelaCuesta, Linda Hurley

**Affiliations:** 1 Center for Alcohol and Addiction Studies, School of Public Health, Brown University, Providence, RI 02903, USA; augustine_kang@brown.edu (A.W.K.); audrey_debritz@brown.edu (A.A.D.); courtney_delacuesta@brown.edu (C.D.); 2 CODAC Behavioral Healthcare Inc., Cranston, RI 02910, USA; mwalton@codacinc.org (M.R.W.); lhurley@codacinc.org (L.H.); 3 College of Public Health, Temple University, Philadelphia, PA 19122, USA; ariel_hoadley@brown.edu

**Keywords:** telehealth services, medication for opioid use disorder, needs assessment, counselors

## Abstract

Using quantitative and qualitative evidence, this study triangulates counselors’ perspectives on the use of telemedicine in the context of Opioid Use Disorder (OUD) treatment. A concurrent mixed-methods design examined counselors’ experiences with telephone counseling during the COVID-19 pandemic. N = 42 counselors who provided OUD counseling services completed a close-ended, quantitative survey examining their experiences in addressing clients’ anxiety, depression, anger, substance use, therapeutic relationship, and substance use recovery using telephone counseling. The survey also assessed comfort, convenience, and satisfaction with telephone counseling. Counselors also completed open-ended responses examining satisfaction, convenience, relationship with patients, substance use, and general feedback with telephone counseling. The synthesis of quantitative and qualitative evidence indicated that a majority of counselors had positive experiences with using telephone counseling to provide services to clients undergoing OUD treatment. Convenience, greater access to clients, and flexibility were among the reasons cited for their positive experience. However, counselors also expressed that the telephone counseling was impersonal, and that some clients may have difficulties accessing appropriate technology for telehealth adoption. Findings suggest that further research with counselors is needed to identify the key elements of an effective integration of telephone counseling with traditional in-person treatment approaches in the post-pandemic era.

## 1. Introduction

The opioid and COVID-19 epidemics have collectively precipitated an increase in opioid-related mortality in 2020 [1]. More than 3500 Emergency Departments (ED) in the United States reported that opioid overdoses consistently increased in 2020 compared to 2019 [2]. Prior to COVID-19, strict state and federal regulations and laws (in the United States) such as The Ryan Haight Act of 2008, the Drug Addiction Treatment Act of 2000, and 42 CFR 8 for opioid treatment programs (OTPs) enforced by the Substance Abuse and Mental Health Service Administration and Drug Enforcement Agency made opioid treatment difficult, time consuming, and costly for patients to access and receive medication and care. These regulations include restrictions on prescribing, dispensing, and limiting take-home medication for opioid use disorder (MOUD), as well as mandated face-to-face medical and clinical encounters to initiate and maintain MOUD [3,4,5,6]. COVID-19 brought about relaxations in laws and regulations, and policies materialized to reduce barriers to treatment reducing financial and administrative obstacles such as prior authorizations, co-pays, and licensing requirements, loosening of take-home MOUD in OTPs; one significant modification was the expansion of telehealth services in opioid use disorder (OUD) treatment. In the final week of March 2020, at the onset of the pandemic, the use of telehealth increased by 154% in the US compared to the same week in 2019 [7].

Behavioral healthcare service providers had to rapidly shift, essentially immediately, to offer virtual services in a way that safeguarded minimal service disruption for individuals in care while having to take on the rising number of new patients remotely [8,9,10].

Telehealth services increase patient access, adherence, and retention in treatment while yielding equivalent outcomes to in-person care [11,12,13,14]. However, rates of telehealth services provision and/or uptake for substance use disorder (SUD) were low before COVID-19 [15]. A systematic review reported that telemedicine-based provision of counseling in SUD was feasible and effective, and a randomized control trial of therapy demonstrated lower attrition among participants receiving telehealth versus in-person care [16,17]. For OUD treatment specifically, some evidence indicates similar rates of counseling attendance, drug-positive urinalysis results, and retention in treatment with telehealth versus in-person-based provision of services [18,19].

While providers are central in building an effective therapeutic relationship, limited literature exists that examines counselor satisfaction or perception of the therapeutic alliance, especially in the context of SUD treatment [20]. A recent survey examining provider satisfaction and telehealth experiences with outpatient mental health treatment providers (including psychiatrists and counselors) reported that providers were largely satisfied with telehealth, but may face challenges with caring for clients with psychiatric comorbidities and those with high symptom severity [21]. In addition, studies have also reported that providers were concerned with security, confidentiality, and technological issues with the use of telemedicine [22,23]. Providers should be cautious when managing patient health information in the context of telemedicine and pay special attention to patient information protections [24]. Other provider apprehensions were unsuitability for specific patients, particularly high-risk suicidal patients [25].

Understanding the experiences of providers is integral to informing the post-pandemic integration of telemedicine in the care provision model for OUD treatment. There are gaps in research regarding providers’ perspectives on the use of telemedicine, particularly in the context of OUD treatment and via the format of telephone counseling. To gain insight into providers’ perspectives on the use of telemedicine for OUD for the purpose of informing post-pandemic policies and care practices, this study aims to both quantitatively and qualitatively examine counselors’ experiences with telephone counseling in the context of OUD treatment provision during the COVID-19 pandemic.

## 2. Materials and Methods

### 2.1. Sample and Data Collection

The current study analyzed data from a survey conducted by CODAC Behavioral Healthcare (Cranston, RI, USA) for a larger quality improvement project to assess counselor experience with telemedicine in the context of COVID-19 risk mitigation. The survey examined the experiences of counselors from seven Opioid Treatment Program (OTP) clinics across Rhode Island who participated in provision of telephone counseling. These counselors provided OUD counseling services at least once a month to their clients. Counselors completed a web-based survey between July and November 2020, and were entered into a raffle for a $25 gift card. The CODAC research oversight committee reviewed and approved the project.

N = 42 counselors completed the online survey which contained 3-point (Less = 1, The Same = 2, and More = 3) and 5-point (Very Dissatisfied = 1 and Very Satisfied = 5) Likert-scale questions and open-ended responses. A total of nine Likert-scale questions examined: (1) Anxiety, (2) depression, (3) anger, (4) substance use, (5) recovery, (6) comfort, (7) convenience, (8) relationship with patients, and (9) overall satisfaction compared to face to face counseling. For example, the anxiety question was phrased as such, “How anxiety is addressed in telephone counseling sessions compared to face to face”. Open-ended responses queried counselors’ experiences with telephone counseling in the following five categories: Satisfaction, convenience, relationship with patients, substance use and recovery, and general feedback. Both the quantitative and qualitative data were collected concurrently.

### 2.2. Concurrent Mixed-Methods Analysis Approach

A concurrent mixed-methods analysis approach was used to analyze the study data [26]. While the quantitative data provides a numerical representation of the study variables (i.e., anxiety and depression), qualitative data additionally provides background and context to those variables by identifying themes and triangulating quantitative findings. This approach provides a comprehensive understanding of the counselors’ experiences with telephone counseling.

In terms of qualitative analysis, the five open-ended response questions were coded using an inductive thematic analysis approach. A codebook was created by two independent coders (J.D. and R.U.); they read the counselors’ open-ended responses and created preliminary codes. A third rater (A.A.D.) applied the codebook to the responses. Emergent themes were discussed, and codes were agreed upon by the authors (A.A.D. and A.W.K.). Twenty two codes were identified within the responses which then were further broken down into thirteen positive valency codes and nine negative valency codes. Participant responses were further grouped into two groups for quantitative analysis: Positive vs. negative/mixed valency. Interrater reliability ranged from 0.78 to 0.92.

For quantitative analysis, binary logistic regression examined the association between the nine close-ended Likert-scale variables and valency groups (Positive = 1, Mixed/Negative = 0). Analysis was performed using IBM SPSS Statistics version 26 [27].

## 3. Results

Table 1 displays the frequencies of the study variables. 32.5% of counselors reported that telephone counseling addressed clients’ anxiety and depression to a lesser extent compared to in-person counseling. 45.9% of counselors reported that telephone counseling addressed anger issues to a lesser extent, and 27.5% of counselors reported that telephone counseling addressed their clients’ substance use and recovery to a lesser extent. In terms of therapeutic relationship variables, 21.4% of counselors reported that telephone counseling was less comfortable compared to in-person counseling, 31% reported that telephone counseling was less convenient, and 9.5% reported that their relationship with their client has worsened during telephone counseling. Overall, 69.0% of counselors were either somewhat or very satisfied with telephone counseling.

Table 2 displays the results of logistic regression, with group membership in the valency variable as the dependent variable (positive = 1, mixed/negative = 0). The following significant associations were observed: (1) A unit increase in substance use recovery score was associated with 5.61 times increased odds of being in the positive valency group (*p* = 0.025). (2) A unit increase in recovery score was associated with 4.36 times increased odds of being in the positive valency group (*p* = 0.036). (3) A unit increase in comfort score was associated with 5.38 times increased odds of being in the positive valency group (*p* = 0.049). (4) A unit increase in convenience score was associated with 8.52 times increased odds of being in the positive valency group (*p* = 0.028). (5) A unit increase in relationship with patients score was associated with 4.81 times increased odds of being in the positive valency group (*p* = 0.037). (6) A unit increase in overall satisfaction score was associated with 4.68 times increased odds of being in the positive valency group (*p* = 0.022).

**Figure 1:**
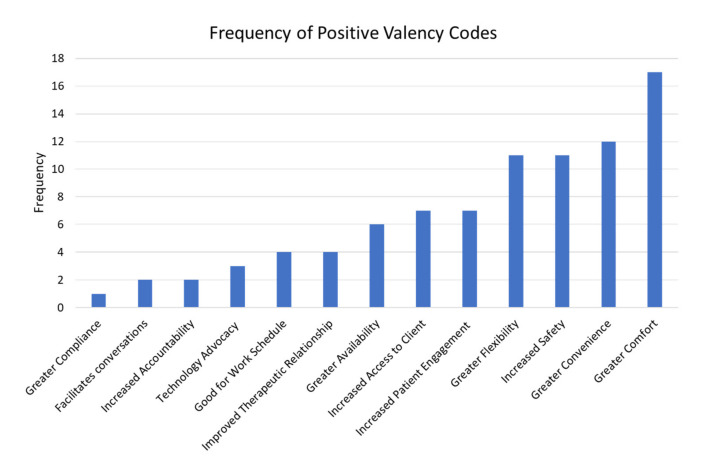
Frequency of positive valency codes.

**Figure 2:**
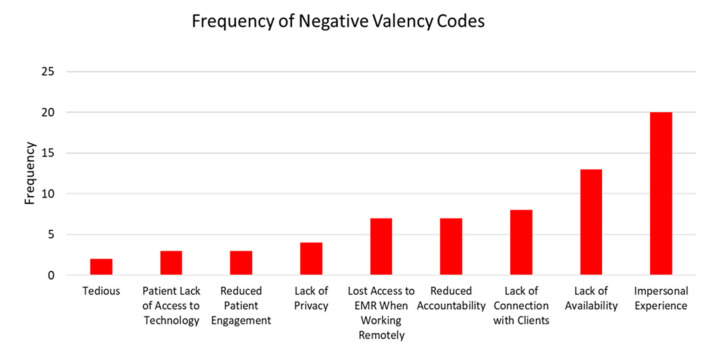
Frequency of negative valency codes.

Table 3 lists an example quote from counselors that corresponds to their coded response. Figure 1 and Figure 2 illustrate the frequencies of the positive and negative valence themes and their codes, respectively.

## 4. Discussion

Our study provides insight into the experiences of licensed counselors who provided telephone counseling services to clients at multiple OTPs during the COVID-19 pandemic. Overall, our results fill key gaps in the literature and indicate a variety of experiences among counselors when providing OUD treatment using telephone counseling.

In general, the majority of counselors had positive experiences with telephone counseling, which was corroborated by their concurrent reports of quantitative and qualitative survey responses. Notably, the convenience of telephone counseling was significantly related to counselors’ overall positive experiences; half of all counselors reported that telephone counseling was more convenient than in-person counseling. The experience of increased convenience was reiterated by counselors in their open-ended responses, where greater convenience was among the most common positive valency codes. In qualitative responses, counselors reported having greater access to their clients, more flexibility, and improved work schedules, each of which could lead to an increased sense of convenience. These findings are consistent with the results of a recent study, which found that both clients and counselors perceive telehealth as convenient [28,29].

Of note, counselors also stated that they believe telephone counseling to be convenient for their clients. Past research has found that inconvenient, in-person appointments are a perceived barrier to clients’ engagement and retention in traditional, in-person counseling [30]. For example, even clients who reside within five miles of a clinic may experience challenges getting to their appointments without a car or have adequate access to public transportation [31]. By comparison, telephone counseling sessions can be completed from home or virtually anywhere, reducing the cost and time of travel to and from the clinic and addressing potential transportation barriers. Counselors appreciated that they had improved access to their clients and some noted that there seemed to be greater client engagement, accountability, and compliance.

Some counselors noted improvements in their therapeutic relationship with their clients after transitioning to a telephone counseling platform. Moreover, counselors also described relational processes, such as increases in comfort level and improved facilitation of conversations, that have previously been positively linked to better therapeutic relationships [31]. In future research, counselor comfort and perceived conversational flow might be studied as potential moderators of the relationship between counseling modality and the quality of the counseling relationship. Past studies have identified the therapeutic relationship as a common factor significantly predictive of client retention and treatment outcomes in counseling [32,33]. While some research found that counselor ratings of the therapeutic relationship were more strongly related to substance use outcomes than clients’ ratings, other researchers have found mixed results [32,34,35]. Thus, it is important to consider multiple perspectives to best understand how the virtual therapeutic experience may relate to client retention, engagement, and treatment outcomes. More attention may be needed to better understand relational processes amongst the majority of counselors who indicated that there was no difference in the therapeutic relationship for telephone counseling versus in-person sessions.

The most common negative valency code from the qualitative responses was an impersonal experience. The inability to build rapport or observe nonverbal cues has been frequently identified as a barrier to telemedicine [36]. Such barriers may also limit the discussion of sensitive topics like depression, anxiety, and anger. This finding is consistent with another study examining mental health providers’ experiences with COVID-19 perpetuated telehealth implementation [21]. Future research should explore ways of addressing this issue, such as by using a hybrid in-person/telehealth approach. In addition, our results suggest that telehealth-based approaches should be mindful of logistical barriers to implementation. For example, the counselors in our study experienced issues with off-site access to the electronic medical record system, with some citing issues with patients’ access to appropriate technology to complete sessions virtually. Hence, training and support are necessary for providers (and possibly patients) to properly adapt to a virtual environment [37].

To our knowledge, there have been no other studies that reported counselor perceptions to telephone counseling implementation in substance use treatment programs. Our study examined only telephonic versions of telehealth services, whereas other studies have observed telehealth implementation of videoconferencing platforms. When provided video platforms, counselors may adapt to telemedicine more favorably, and mental health and substance use clinics have reported benefits to the continuity of patient care, both of which align with the findings of our study [37,38].

There were several limitations in our study. In terms of providers, we only collected survey data from OTP counselors (and did not include prescribers such as physicians), Due to our cross-sectional design, we were unable to assess changes in counselor perspectives (e.g., it is unclear if fatigue with the pandemic impacted perspectives towards telehealth counseling). We also did not assess the counselors’ attitudes and acceptability of working remotely, which may have confounded their responses. In addition, it is unclear if the quality of therapeutic alliance between counselors and their patients affected counselors’ responses; future research should examine the impact of therapeutic alliance on counselors’ perspectives toward telehealth service provision. Lastly, our study sample was geographically limited to Northeastern USA, and results may not be generalizable to other regions and countries.

## 5. Conclusions

The rapid transition of counseling to telehealth platforms has raised the question of whether telemedicine, such as telephone counseling, should be further incorporated into post-pandemic healthcare services and the best practices for implementation. The success and sustainability of a telehealth program will be highly dependent on counselor buy-in. Prior to the onset of the pandemic and the rapid implementation of telehealth treatment, knowledge on the effectiveness of virtual counseling compared to face-to-face office visits was limited. Our findings suggest that further research with counselors is needed to identify the key elements of effective integration of telephone/virtual counseling with traditional in-person treatment approaches in the post-pandemic era.

## Figures and Tables

**Table 1:** Frequencies for Anxiety, Depression, Anger, Substance, Recovery, Comfort, Convenience, Relationship with Patients, and Overall Satisfaction.

Predictor	*n*	Less	The Same	More
Anxiety	40	13 (32.5)	18 (45.0)	9 (22.5)
Depression	40	13 (32.5)	23 (57.5)	4 (10.0)
Anger	37	17 (45.9)	16 (43.2)	4 (10.8)
Substance	40	11 (27.5)	22 (55.0)	7 (17.5)
Recovery	40	11 (27.5)	21 (52.5)	8 (20.0)
Comfort	42	9 (21.4)	28 (66.7)	5 (11.9)
Convenience	42	13 (31.0)	8 (19.0)	21 (50.0)
Relationship with Patients	42	4 (9.5)	25 (59.5)	13 (31.0)
		Very Dissatisfied/Dissatisfied	Neither	Somewhat/Very Satisfied
Overall Satisfaction with Telephone Counseling	42	5 (11.9)	8 (19.0)	29 (69.0)

Note. Data expressed as *n* (%).

**Table 2:** Logistic regression results comparing study variables with valency (Positive = 1, Mixed/Negative = 0).

Predictor	M ± SD	B	SE	Wald	*df*	*p*	Exp(B)	95% CI of Exp(B)
Anxiety		0.310	0.544	0.325	1	0.569	1.363	(0.470, 3.955)
Positive (*n* = 8) Mixed/Negative (*n* = 30)	2.00 ± 0.76 1.83 ±.75							
Depression		0.499	0.678	0.541	1	0.462	1.646	(0.436, 6.218)
Positive (*n* = 8) Mixed/Negative (*n* = 30)	1.88 ± 0.64 1.70 ± 0.60							
Anger		0.850	0.744	1.306	1	0.253	2.339	(0.545, 10.050)
Positive (*n* = 6) Mixed/Negative (*n* = 29)	1.83 ± 0.41 1.52 ± 0.63							
Substance		1.725	0.768	5.049	1	0.025 *	5.611	(1.246, 25.255)
Positive (*n* = 8) Mixed/Negative (*n* = 30)	2.38 ± 0.74 1.73 ± 0.58							
Recovery		1.472	0.703	4.382	1	0.036 *	4.360	(1.098, 17.306)
Positive (*n* = 8) Mixed/Negative (*n* = 30)	2.38 ± 0.74 1.77 ± 0.63							
Comfort		1.683	0.859	3.845	1	0.049 *	5.384	(1.001, 28.971)
Positive (*n* = 9) Mixed/Negative (*n* = 30)	2.22 ± 0.44 1.77 ± 0.57							
Convenience		2.143	0.973	4.854	1	0.028 *	8.523	(1.267, 57.366)
Positive (*n* = 9) Mixed/Negative (*n* = 30)	2.89 ± 0.33 1.90 ± 0.88							
Relationship with Patients		1.571	0.754	4.339	1	0.037 *	4.812	(1.097, 21.105)
Positive (*n* = 9) Mixed/Negative (*n* = 30)	2.56 ± 0.53 2.07 ± 0.58							
Overall Satisfaction		1.543	0.672	5.269	1	0.022 *	4.677	(1.253, 17.457)
Positive (*n* = 9) Mixed/Negative (*n* = 30)	4.44 ± 0.53 3.47 ± 1.07							

Note. * represents statistical significance.

**Table 3:** Examples of quotes for coded responses.

Example Quote	Name of Code
Positive Valency Theme	
*More convenient as patients who are typically rushed to dose and leave (because of transportation or whatnot)*	Convenience
*I think some of the patients are better able to express themselves without having the feeling of being stared at or judged in some way in an office setting.*	Facilitates Conversations
*It is easier for patients who work early or receive rides to the clinic to complete sessions, as there is more flexibility. Patients seem to be more engaged in sessions because they are not feeling rushed.*	Good for Work Schedule
*Patients are more comfortable with phone sessions and availability is better.*	Greater Availability
*I think it has provided more comfort to clients and easier access to clinical staff if necessary compared to counseling requirements needing to be completed in person.*	Greater Comfort
*I get more compliance with sessions with some patients/clients.*	Greater Compliance
*i am able to coordinate session times with more flexibility throughout the day rather than being dependent on the dosing hour window*	Greater Flexibility
*Keeping in touch with patients when they are outside the clinic has helped a lot of patients who have little/no supports otherwise. I feel that my rapport with my clients has increased substantially over the past several months due to telephonic counseling.*	Improved Therapeutic Relationship
*… I’ve also had the opportunity to touch base with my patients more frequently since we’re not not constrained to dosing schedules (Ex. maybe only in the clinic 2–4 times per month) and we don’t have to juggle multiple patients waiting to be seen when they come to the clinic.*	Increased Access to Client
*For some patients it is easier for them to take accountability for their use over the telephone and work on recovery supports and treatment goals.*	Increased Accountability
*Patients are more engaged in phone sessions and are not in a rush to end session.*	Increased Patient Engagement
*Tele-health calling is a way to contact patients and provide safe effective measure of counseling and release for our patients. Allowing them to remain at a safe distance for all involved.*	Increased Safety
*… I think in the future when Covid-19 is no longer a major threat and we do face to face office visits we should keep either telephone, or Zoom individual sessions also in case someone has no ride or we have a snow day etc to stop having to cancel sessions.*	Technology Advocacy
**Negative Valency Theme**	
*I don’t think telephone counseling sessions are as valuable as face-to-face counseling.*	Impersonal Experience
*I don’t mind doing telehealth sessions, but it is a lot harder to reach patient’s. I feel like I am always scrambling near the end of the month to see people who don’t answer the phone.*	Lack of Availability
*…patients often have limited privacy, this is amplified by patients children and family member being at home more often due to COVID-19 resulting in more adults and children being at home due to loss of employment and distance learning for students…*	Lack of Privacy
*All services provided would have been more manageable with off site access to SMART system. Moving forward should tele counseling continue, off site access would enable counselors to complete documentation more thoroughly in order to manage their caseload more effectively. While also promoting better communication between staff.*	Lost Access to EMR When Working Remotely
*The counselor has to be straight forward and ask are you depressed? how is your anxiety? are you using any substances? Its really the same on the phone.*	No Difference
*phones not working, voice mail box is full, patients cannot always pay phone bill on time, patients don’t always check voice mail*	Patient Lack of Access to Technology
*Lessens rapport, accountability, structure and consistency. All essential in the road to recovery and abstinence.*	Reduced Accountability
*I think the conversations telephonically can be just as supportive as face to face; however some patients do not engage in phone conversations as well as in person.*	Reduced Patient Engagement
*It works on a level but then it does not. It was fine at first but now it is very tedious to do the phone calls*	Tedious
*I think overall it is harder to provide clinical care as effectively over the phone as it would be in person. Body language and facial expressions can give us a lot of information, it can make it more difficult not having that in front of us to observe. However, given the circumstances I think it is the best alternative we have available.*	Telephone Counseling Lacks Communication/Connection With Clients

## Data Availability

The data presented in this study are available on request from the corresponding author. The data are not publicly available due to privacy and ethical considerations.
